# Supplementary material for: Characterization of T cell responses to co-administered hookworm vaccine candidates Na-GST-1 and Na-APR-1 in healthy adults in Gabon
Source: PLoS Negl Trop Dis. 2021 Oct 1;15(10):e0009732. doi: 10.1371/journal.pntd.0009732 (PMC8486127; doi:10.1371/journal.pntd.0009732)
Supplement: S1 Table — (DOCX) [file pntd.0009732.s001.docx]

| **Antibody** | **Label** | **Compagny** | **Catalogue number** | **Clone** | **Dilution** |
| --- | --- | --- | --- | --- | --- |
| **IL-2** | FITC | BD Biosciences^a^ | 340448 | 5.344.111 | 25x |
| **IL-4** | PE | BD Biosciences | 340451 | 3.010.211 | 20x |
| **IL-5** | PE | Biolegend^b^ | 504303 | TRFKJ | 250x |
| **IL-13** | PE | Biolegend | 501903 | JES10-5A2 | 100x |
| **CD4** | PerCP- eF710 | eBiosciences^c^ | 46-0047-41 | SK3 | 400x |
| **TNF-α** | PE-CY7 | eBiosciences | 25-7349-41 | Mab11 | 1000x |
| **IL-10** | APC | BD Biosciences | 554707 | JES3-19F1 | 250x |
| **CD3** | APC- eF780 | eBiosciences | 47-0038-41 | UCHT1 | 800x |
| **IFN-γ** | BV-421 | Biolegend | 502531 | 4SB3 | 1000x |
| **LIVE/DEAD** | Aqua | Life technology^d^ | L34957 |  | 400x |
| **FcR inhibitor (CD16/32/64)** | | eBiosciences | 14-9161 |  | 100x |

^a^BD Bioscence, San Jose, CA, USA. ^b^Biolegend, San Diego, CA, USA. ^c^eBiosciences, San Diego, CA, USA. ^d^Life technology, Carlsbad, CA, USA. CD, cluster of differentiation. FITC, fluorescein isothiocyanate. PE, R-phycoerythin. APC, allophycocyanin.
