# Supplementary material for: Characterization of T cell responses to co-administered hookworm vaccine candidates Na-GST-1 and Na-APR-1 in healthy adults in Gabon
Source: PLoS Negl Trop Dis. 2021 Oct 1;15(10):e0009732. doi: 10.1371/journal.pntd.0009732 (PMC8486127; doi:10.1371/journal.pntd.0009732)
Supplement: S2 Table — (DOCX) [file pntd.0009732.s002.docx]

| **Label** | **Markers** | **Clone** | **Company** | **Catalogue Number** | **Dilution** |
| --- | --- | --- | --- | --- | --- |
| ^089^Y | CD45 | HI30 | Fluidijm^a^ | 3089003B | 200x |
| ^113^Cd | CD45RA | HI100 | eBiosciences^b^ | 83-0458-42 | 50x |
| ^115^In | CD57 | HNK-1 | Biolegend^c^ | 359602 | 100x |
| ^141^Pr | CD196 (CCR6) | G034E3 | Fluidijm | 3141003A | 100x |
| ^142^Nd | CD19 | HIB19 | Fluidijm | 3142001B | 200x |
| ^143^Nd | CD117 (c-kit) | 104D2 | Fluidijm | 3143001B | 100x |
| ^144^Nd | IL-2 | MQ117H12 | Biolegend | 500339 | 400x |
| ^145^Nd | CD4 | RPA-T4 | Fluidijm | 3145001B | 100x |
| ^146^Nd | CD8a | RPA-T8 | Fluidijm | 3146001B | 200x |
| ^147^Sm | CD183 (CXCR3) | G025H7 | Biolegend | 353733 | 100x |
| ^148^Nd | CD14 | M5E2 | Biolegend | 301843 | 100x |
| ^149^Sm | CD25 (IL-2R) | 2A3 | Fluidijm | 3149010B | 100x |
| ^150^Nd | CD185 (CXCR5) | RF8B2 | Biolegend | 356902 | 100x |
| ^151^Eu | CD278 (ICOS) | C398.4A | Biolegend | 313502 | 50x |
| ^152^Sm | TCRgd | 11F2 | Fluidijm | 3152008B | 50x |
| ^153^Eu | CD7 | CD7-6B7 | Fluidijm | 3153014B | 100x |
| ^154^Sm | CD154 (CD40L) | 24-31 | Biolegend | 310835 | 100x |
| ^155^Gd | IFN-γ | B27 | Biolegend | 506521 | 400x |
| ^156^Gd | CD294 (CRTH2) | BM16 | Biolegend | 350102 | 50x |
| ^158^Gd | CD122 | Tu27 | Biolegend | 339015 | 100x |
| ^159^Tb | CD197 (CCR7) | G043H7 | Fluidijm | 3159003A | 100x |
| ^160^Gd | TNF-α | Mab11 | Biolegend | 502941 | 400x |
| ^161^Dy | KLRG1 (MAFA) | 14C2A07 | Miltenyi^d^ | Special order | 100x |
| ^162^Dy | CD11c | Bu15 | Fluidijm | 3162005B | 200x |
| ^163^Dy | CD152 (CTLA-4) | BNI3 | Biolegend | 369602 | 100x |
| ^164^Dy | CD161 | HP-3G10 | Fluidijm | 3164009B | 100x |
| ^165^Ho | CD127 (IL-7Rα) | A019D5 | Fluidijm | 3165008B | 200x |
| ^166^Er | IL-10 | JES39D7 | Fluidijm | 3166008B | 400x |
| ^167^Er | CD27 | O323 | Fluidijm | 3167002B | 200x |
| ^168^Er | HLA-DR | L243 | Biolegend | 307651 | 200x |
| ^169^Tm | IL-4 | MP4-25D2 | Fluidijm | 3169016B | 400x |
| ^169^Tm | IL-5 | TRFK5 | Biolegend | 500829 | 400x |
| ^169^Tm | IL-13 | JES105A2 | Biolegend | 504309 | 400x |
| ^170^Er | CD3 | UCHT1 | Fluidijm | 3170001B | 100x |
| ^171^Yb | CD28 | CD28.2 | Biolegend | 302937 | 200x |
| ^172^Yb | CD38 | HIT2 | Fluidijm | 3172007B | 200x |
| ^173^Yb | CD45RO | UCHL1 | Biolegend | 304239 | 100x |
| ^175^Lu | CD279 (PD-1) | EH12.2H7 | Fluidijm | 3175008B | 100x |
| ^176^Yb | CD56 | HCD56 | Fluidijm | 3176008B | 100x |
| ^198^Pt | CD86 | BU63 | Biolegend | 305435 | 100x |
| ^209^Bi | CD16 | 3G8 | Fluidijm | 3209002B | 200x |

^a^Fluidigm, South San Francisco, CA, USA. ^b^eBioscience, San Diego, CA, USA. ^c^Biolegend, San Diego, CA, USA. ^d^Miltenyi Biotech, Bergisch Gladbach, Germany. CCr, C-C chemokine receptor. CD, cluster of differentiation. CRTH2, prostaglandin D2 receptor 2. CXCR, CXC chemokine receptor. HLA-DR, human leukocyte antigen-D-related. IL-2R, interleukin-2 receptor. IL7Rα, interleulin- 7 receptor α. KLRG1, killer cell lectin-like receptor subfamily G member 1. MAFA, mast cell function-associated antigen. PD-1, programmed cell death protein. TCR, T-cell receptor. Markers in grey were stained intracellular, while all other markers were stained on the cell surface.
