# Supplementary figures and images for: Characterization of T cell responses to co-administered hookworm vaccine candidates Na-GST-1 and Na-APR-1 in healthy adults in Gabon
Source: PLoS Negl Trop Dis. 2021 Oct 1;15(10):e0009732. doi: 10.1371/journal.pntd.0009732 (PMC8486127; doi:10.1371/journal.pntd.0009732)

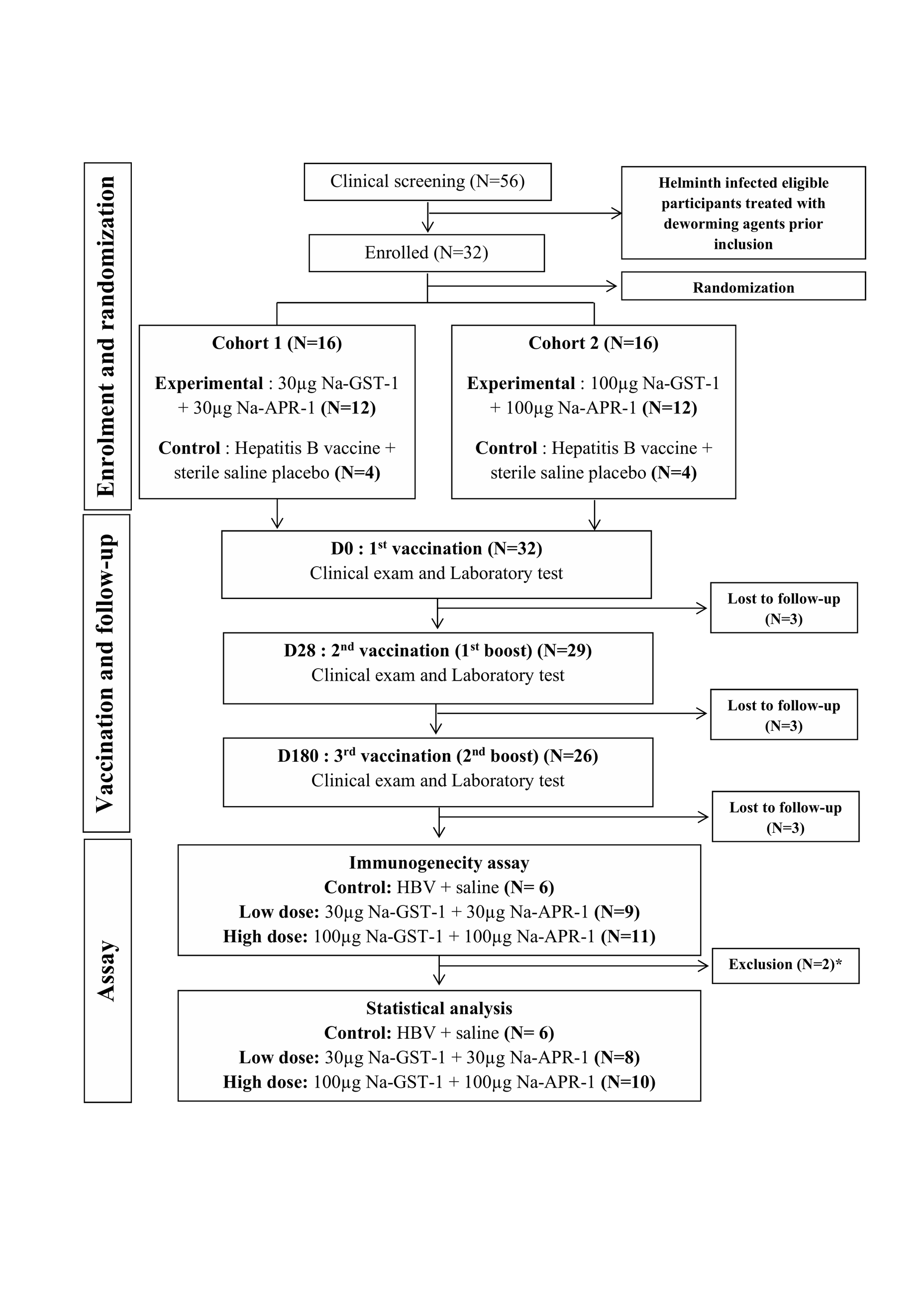

Supplement: S1 Fig — (TIF) [file pntd.0009732.s003.tif]

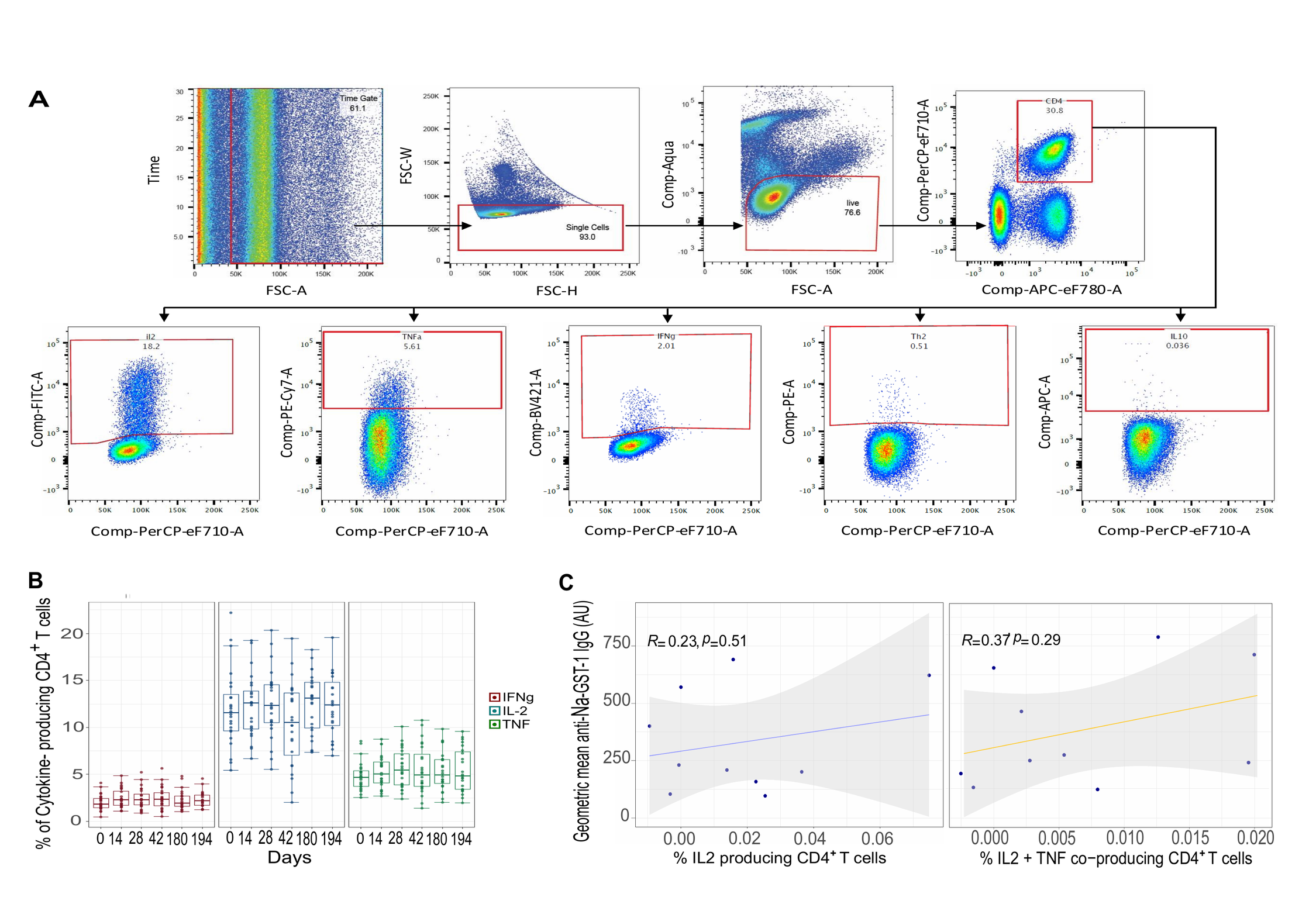

Supplement: S2 Fig — (A) Flow cytometry gating strategy of CD4+ T cells for one representative sample after SEB stimulation. Cells were first gated on time or Forward scatter (FSC)-W and FSC-H to select singlets. Thereafter, cells were gated on viability dye and FSC-A to select live cells. Subsequently, cells producing IL-2, TNF, IFN-γ, IL-10 and the TH2 cytokines (IL-4, IL-5 and IL-13) were gated from cells expressing CD3 and CD4. (B) Cytokine response to SEB over time. Boxplots representing the median, 1st and 3rd quantile. Whiskers are extending to the maximum/minimum, no further than 1.5x the IQR. All points are shown. (C) Spearman correlation between the change (day 194 –day 0) induced by vaccination in Na-GST-1 specific IgG antibody levels, given in arbitrary units (AU), and the frequency of IL-2 producing CD4+ T cells, in the high dose group. Solid line indicates predicted values from linear regression analysis with the shaded band showing the 95% confidence interval. (TIF) [file pntd.0009732.s004.tif]

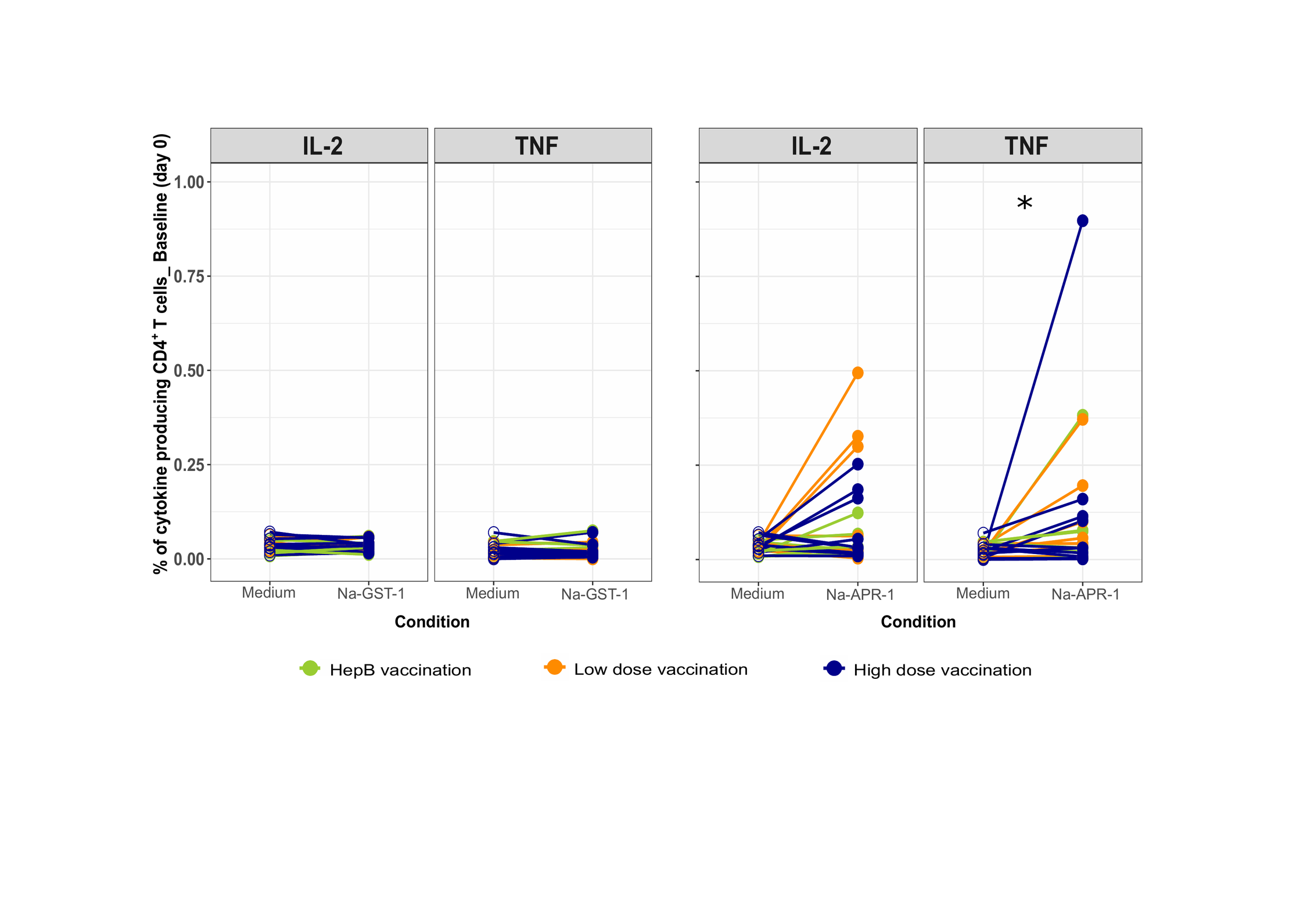

Supplement: S3 Fig — The frequency of IL-2 and TNF producing CD4+ T cells in response to Na-GST-1 or Na-APR-1 stimulation compared to medium at baseline (day 0), in control (green line), low dose (orange line) and high dose group (blue line). Cells producing cytokines are expressed as percentage of CD4+ T cells. Each line represents an individual donor. Wilcoxon one-tailed paired test was performed for comparison between medium and Na-GST-1 or Na-APR-1 stimulation. (*) indicates the significance p≤0.05 in (A) and (B). (TIF) [file pntd.0009732.s005.tif]

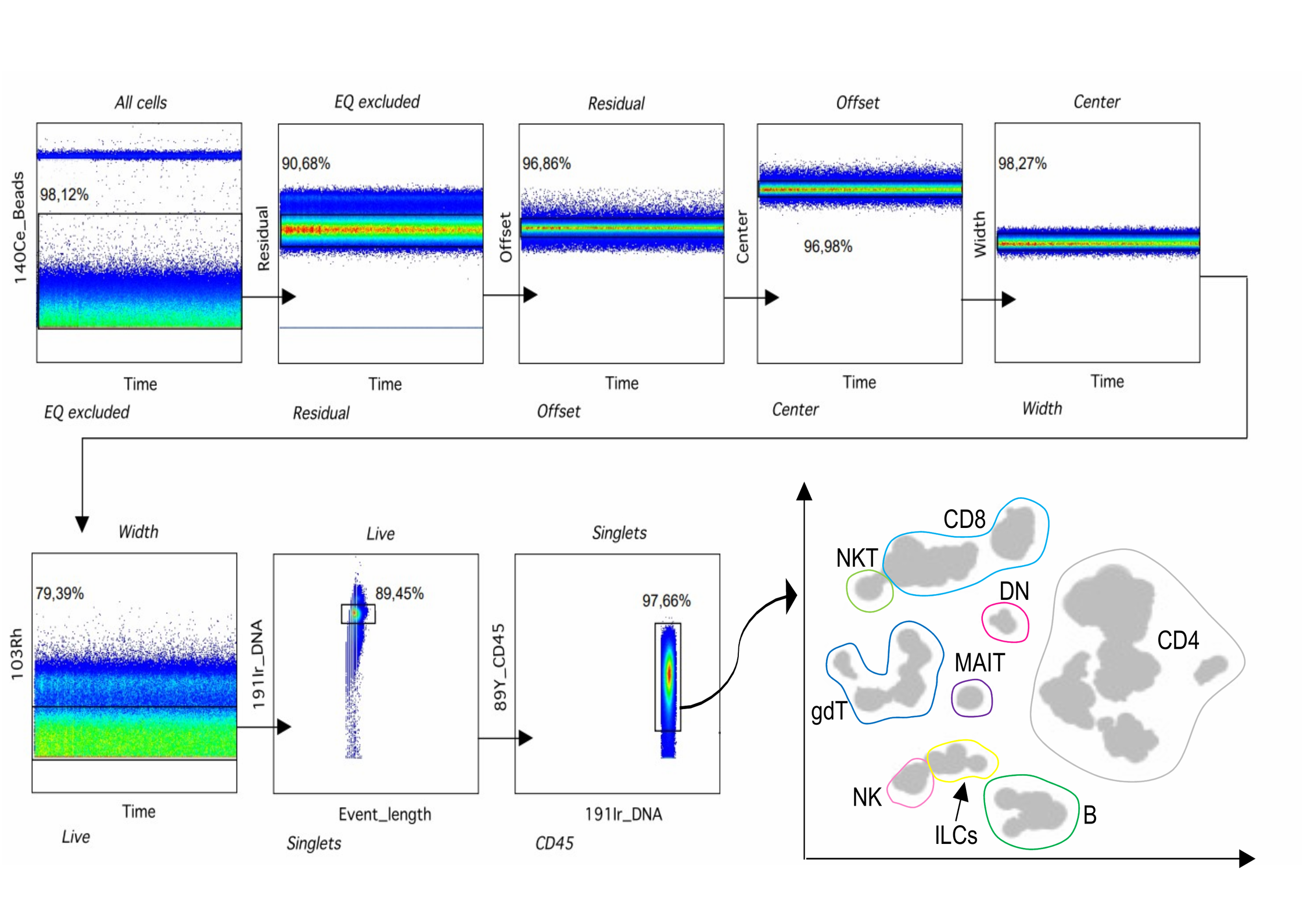

Supplement: S4 Fig — Unsupervised analysis was performed using HSNE on Cytosplore to visualize the relative distribution of cell populations within the CD45+ cell. At the first level HSNE embedding was used where CD45+ cells clustered based on surface markers expression, identifying the major immune lineages were identified. (TIF) [file pntd.0009732.s006.tif]

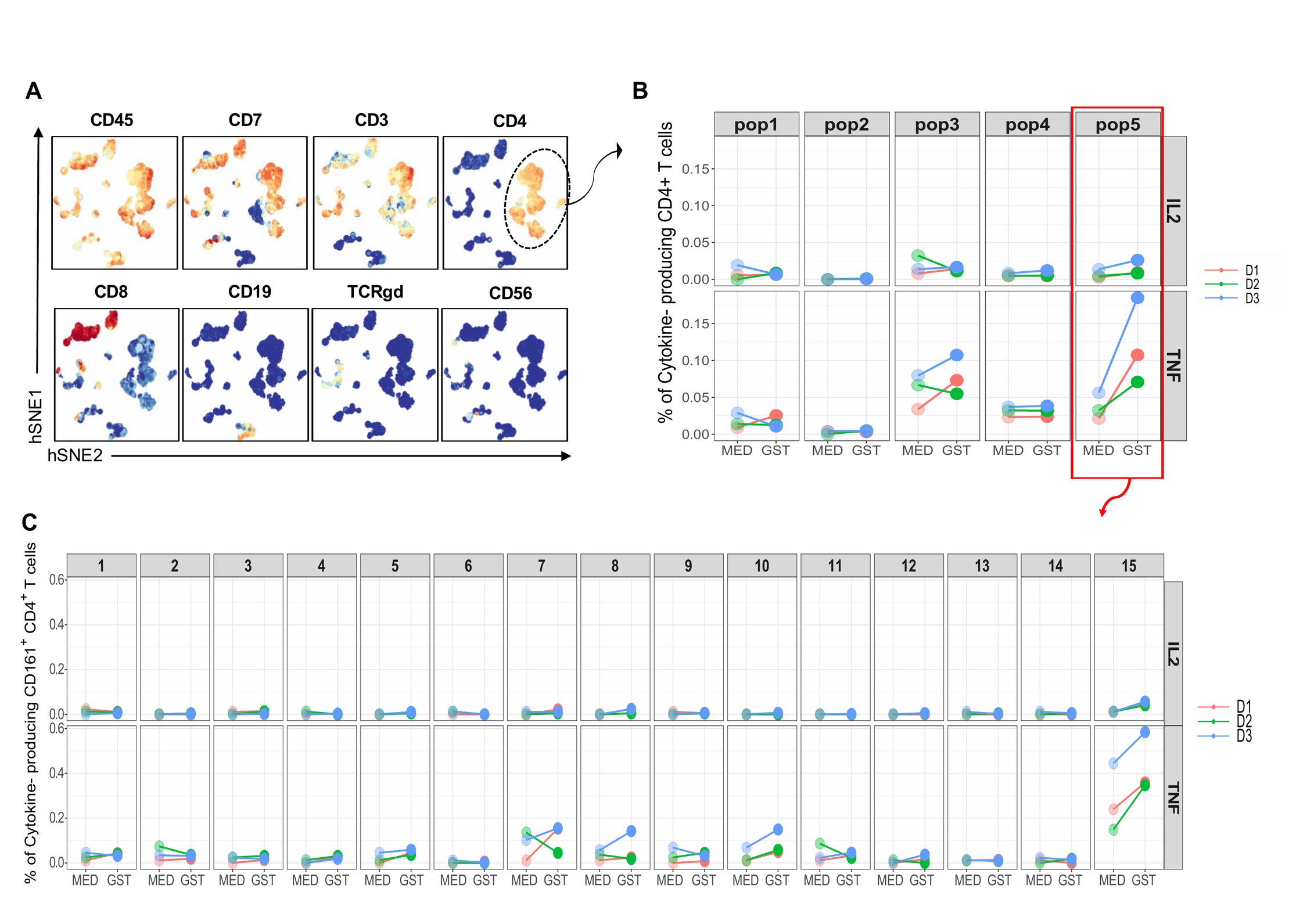

Supplement: S5 Fig — (A) HSNE embedding depicting the marker expression of the major immune lineages identified within CD45+ cells. The colour represents the arcsin5- transformed expression values of indicated markers. (B) Cytokines producing CD4+ T cell populations in Na-GST-1 stimulated cells. The frequency of cytokine producing cells is of total number of CD4+ T cells. (C) Cytokines producing CD161+ CD4+ T cell population in Na-GST-1 stimulated cells in all donors. The frequency of cytokine producing is of total number of CD161 expressing CD4+ T cell. Both medium (MED) and Na-GST-1 (GST) stimulation is shown and lines depict the same donors in (B) and (C). (TIF) [file pntd.0009732.s007.tif]
