# Supplementary material for: Characterization of T cell responses to co-administered hookworm vaccine candidates Na-GST-1 and Na-APR-1 in healthy adults in Gabon
Source: PLoS Negl Trop Dis. 2021 Oct 1;15(10):e0009732. doi: 10.1371/journal.pntd.0009732 (PMC8486127; doi:10.1371/journal.pntd.0009732)
Supplement: Attachment — Submitted filename: Response to Reviewers.docx [file pntd.0009732.s008.docx]

**Characterization of T cell responses to co-administered hookworm vaccine candidates *Na*-GST-1 and *Na*-APR-1 in healthy adults in Gabon.**

Yoanne D. Mouwenda^†1,2^, Madeleine E. Betouke Ongwe^*1,2,3^, Friederike Sonnet^*2^, Koen A. Stam^2^, Lucja A. Labuda^2^, Sophie De Vries^4^, Martin P. Grobusch^1,6,7^, Frejus J. Zinsou^1,6^, Yabo J. Honkpehedji^1,6^, Jean C.D. Agobe^1,6^, David J. Diemert^5^, Remko van Leeuwen^7^, Maria E. Bottazzi^8^, Peter J. Hotez^8^, Peter G. Kremsner^1,6,9^, Jeffrey M. Bethony^5^, Simon P. Jochems^2^, Ayola A. Adegnika^1,2,6,9^, Marguerite Massinga Loembe^1^, Maria Yazdanbakhsh^2^

**Responses to reviewer’s comments**

**Reviewer #1**:

Method:

1- Sufficient information should be provided on subject selection an immunization without having to read reference 21. A flow diagram would be useful showing randomizations (N=32) and losses to follow-up yielding number finally analysed (N=24). Figure 1, although useful, is not helpful in this respect because it does not show randomizations (allocated to what?) and shows the number followed-up rather than the number randomized with the final ‘3-group’ composition (presumably the groups to which subjects were randomized?).

**Response**: A flow diagram has been added as supplementary (Appendix S1) that shows randomization and the lost to follow-up that leads to the final number of participants analysed in this study, as shown below.

2- I am curious why the subjects were vaccinated intramuscularly in the forearm (line 129) rather than the upper arm where the deltoid muscle is found?

**Response**: Apologies! It was a mistake, the vaccines were indeed administered intramuscularly in the upper arm (in the deltoid muscle) on study days 0, 28, and 180. This has now been corrected in the manuscript (page 6, line132-133).

3- (line 141): What is ‘(M74)’?

**Response:** To improve the stability of the *Na*-APR1, two aspartic acid residues were mutated to alanines to make *Na*-APR-1 (M74), which is a catalytically inactivated mutant protein. This, recombinant *Na*-APR-1 (M74) was used in the vaccine as well as in the in vitro assays.

This information has been added to the manuscript (page 6, line 126-129 ).

4- (line 148): did the Frosty™ Freezing Container contain isopropyl alcohol?

**Response**: Indeed, the Frosty™ Freezing Container contained isopropyl alcohol. This has now been added to the manuscript (page 7, line 152).

5- (line 207): 5*10^5^ = 5x10^5^?

**Response**: yes 5*10^5^ = 5x10^5^. It has been edited accordingly (page 9, line 214).

6- (line 209): 500x diluted?

**Response**: yes. The co-stimulatory antibodies aCD28 and aCD49d were diluted 500 times. Now the final concentration has been indicated (page7, line 167 and page 9, line 217).

7- (statistical analysis) – this needs to be edited carefully for clarity.

**Response**: The statistical analysis has been edited as shown below and now indicated on page 9 and 10 and starting from line 224.

“For each stimulation condition, the percentages of CD4^+^ T cells producing each cytokine or cytokine combination were determined. The response to stimulation with medium was subtracted from the Ag stimulated responses to give us antigen specific percentage of cytokine producing cells. Linear mixed-effects models [27] were used to assess the change in cytokine producing CD4^+^ T cells over time, in the vaccinated groups and control. The participant ID was modeled as random effect, the time as fixed effect and the percentage for cytokine producing cells as the dependent variable. To assess the change between the frequency of CD4^+^ T cells producing one or more cytokines at day 194 compared to baseline, pairwise comparisons were performed using Wilcoxon paired test. The statistical test was one-tailed with significance level set at p ≤ 0.05. Spearman’s correlation was used to determine the correlation between percentages of CD4+ T cells producing cytokines and the antibody response. Krushal Wallis test was used to compare TNF production following anti-CTLA-4 blocking with isotype and medium conditions.. All statistical analysis were performed in R software [28].”

Results: (No comments)

Conclusion: Yes, conclusions supported by data presented

Abstract:

1- It would be useful to provide more information on methods in the abstract: x (32 or 24?) Gabonese volunteers were randomized to vaccination with three doses of a low-dose (n=8) or high-dose (n=10) combination Na-GST-1 or Na-APR-1 or Hepatitis B vaccine (n=6) over a 6-month period. Blood was collected pre-vaccination on days 0, 28, and 180 and 2-weeks after each vaccine dose on days 14, 42, and 194. PBMCs were isolated from blood samples…etc.
2- would be good to include some relevant data?

**Response**: The abstract has been edited accordingly and is appended below:

“Two hookworm vaccine candidates, *Na*-GST-1 and *Na*-APR-1, formulated with Glucopyranosyl Lipid A (GLA-AF) adjuvant, have been shown to be safe, well tolerated, and to induce antibody responses in a Phase 1 clinical trial (Clinicaltrials.gov NCT02126462) conducted in Gabon. Here, we characterized T cell responses in 24 Gabonese volunteers randomized to vaccination with three doses of 30µg (n=8) or 100µg (n=10) of *Na*-GST-1 or *Na*-APR-1 or Hepatitis B vaccine (n=6) over a 6-month period. Blood was collected pre- and post-vaccination on days 0, 28, and 180 as well as 2-weeks after each vaccine dose on days 14, 42, and 194 for PBMCs isolation. PBMCs were stimulated with recombinant *Na*-GST-1 and *Na*-APR-1, before (days 0, 28 and 180) and two weeks after (days 14, 42 and 194) each vaccination and used to characterize T cell responses by flow and mass cytometry. A significant increase in *Na*-GST-1 -specific CD4^+^ T cells producing IL-2 and TNF, correlated with specific IgG antibody levels, after the third vaccination (day 194) was observed. In contrast, no *Na*-APR-1 specific T cell responses could be detected. Mass cytometry revealed that, *Na*-GST-1 cytokine producing CD4^+^ T cells were CD161^+^ memory cells expressing CTLA-4 and CD40-L. Blocking CTLA-4 enhanced the cytokine response to *Na*-GST-1.

In Gabonese volunteers, hookworm vaccine candidate, *Na*-GST-1, induces detectable CD4^+^ T cell responses that correlate with specific antibody levels. As these CD4^+^ T cells express CTLA-4, and blocking this inhibitory molecules resulted in enhanced cytokine production, the question arises whether this pathway can be targeted to enhance vaccine immunogenicity.

Introduction:

1- Lines 114-115: Please correct: “(Ascaris lubricoides, Trichuris trichura, Necator ankylostoma and Strongiloides stercoralis)”

**Response**: It has been edited accordingly (page 5, line 114-115).

**Reviewer #2**:

Methods: The methods are sound and well written. There is no ethics or regulatory concerns.

Results: The paper is overall clear and well written, but sometimes lack in details in the description of results.

1- For example regarding Figure 2, the authors state : "Following vaccination *Na*-GST-1-specific responses were detectable, but *Na*-APR-1-specific responses were not.” This is a broad statement, especially as the first sentence of the paragraph. Could the author better explain their results? Is it that restimulation with *Na*-APR-1 did not give any T cell response at all, or no specific cytokine response. which cytokines have been tested? Here the negative result is actually quite important and should be further defined.

**Response**: Restimulation with *Na*-APR-1 showed significant detectable TNF producing CD4^+^ T cells response at baseline as illustrated in S5 Appendix (see below). However, *Na*-APR-1 response did not increase following vaccination. Manuscript discussion paragraphs have been edited for precision (page 15, line 354-362)

2- Have the authors considered measuring total cytokines released in the medium after stimulation, as it might be more sensitive that intracellular detection.

**Response:** Although the reviewer is correct to ask for cytokines released, as this is a more sensitive method to detect cytokines following PBMC stimulation, we were interested in cytokines produced by CD4 T cells to assess the levels of T cell help that was induced by the vaccine for antibody production. We present data that TNF+CD4+ T cells were correlated with antibody levels induced.

3- Figure 2, in the legend on the figure orange is indicated as GST 30 ug and blue for GST 100ug. To my understanding all participants have been immunized with both vaccine candidates (Na-GST-1 and Na-APR-1). It is unclear thus if the colour refer to the immunisations or whether it refer to restimulation of the PBMC in vitro.

**Response**: The colours refer indeed to *Na*-GST-1 restimulation of the PBMC in vitro. Orange shows the response to *Na*-GST-1 in subjects vaccinated with low dose and blue, the response in subjects vaccinated with high dose . The figure 3 legend has been adjusted for clarity.

4- (Lines246-247): the authors state “No significant changes were detected when considering low dose Na-GST-1vaccinated group (Fig 2A)”. Is it compared to day 0 (baseline) or to the other groups (HBV or NA-GST-1 100ug).

**Response**: The change in cytokine responses is relative to baseline (day 0) within a particular group, in this case the low dose group. Text has been adjusted to make this clear (page 12, line 270-271).

5- (Line 289) : There is no letter D in Figure S4

**Response**: The mistake has been corrected in the manuscript.

6- Fig 3 D while quite standard, really poor choice of colour for the level of expression as the cluster 15 appears in white and can barely be seen in the image. Maybe the authors could use black rather than white in the middle of the gradient to facilitate visualization of the results.

**Response**: The colour palette is implemented by the software cytosplore and cannot be adjusted for only one colour. It always gives a spectrum. We have tried several options but it does not improve visualization. In addition, the colour scheme is in line with the Figure 3A. However the background colour of the picture was changed to black as suggested by reviewer for more clarity.

7- Figure S4 The mass cytometry panel is quite comprehensive and allow detection of numerous populations. While the authors might want to keep some of the exploitation of this work for future publications, a few interesting questions could enhance the current study:
   - Has the day 194 been compared to any other time point, such as the baseline ? Is there any changes observed in any population?

**Response:** The flow cytometry data showed that CD4^+^ T cells were the main cytokine producers in response to *Na*-GST-1 stimulation and that the response increased (statistically significantly) only at day 194 compared to baseline and at no other time points. No other time points were therefore considered for the mass cytometry analysis. Mass cytometry analysis confirmed that CD4^+^ T cells were the main cytokine producing cells in response to *Na*-GST-1 stimulation. B cells were also seen to produce TNF in response to *Na*-GST-1 in all donors examined. However as only day 194 was considered, we cannot say anything about the change in TNF producing B cells over the vaccination course. The idea here was to characterise, in depth, the responding cells. Further studies will be needed to look at the changes with time as pointed out by the reviewer. This is the first step to show that we can use CyTOF to characterize Ag-specific cells.

8- It would indeed be interesting to understand whether the characteristics of the memory T cells here, results from a previous infection with hookworms (other endemic pathogen) or whether it results from the vaccination.

**Response:** That is an important point. All volunteers come from hookworm endemic area. If we consider the group vaccinated with HBV, we do not see an increase in memory cytokine producing CD4^+^ T cells over time. Suggesting that the increase seen in the high dose group is linked to the vaccination. Regarding whether recent hookworm infection would affect memory responses, we do not have sufficient data to conclude anything. None of the donors used for the CyTOF assay were positive for hookworm infection prior to inclusion, which does not allow any conclusions to be drawn.

9- CTLA-4 might be high regardless of the vaccination with Na proteins. What happens in the HBV control group.

**Response**: HBV control group was not included in the CyTOF analysis as the flow cytometry data showed no increase in *Na*-GST-1- specific response from baseline to day 194. However, as reviewer mentions, it would be interesting to also characterize the HVB specific T cells following vaccination to see if CTLA-4 is also increased ie a general response of T cells to vaccination.

10- Do the results confirm the results from figure 2, is the sensitivity/resolution any better in particular for the type 2 cytokines. One might wonder whether stimulating with APR-1 as well might be interesting.

**Response**: The mass cytometry results confirm the results from figure 2. Type 2 cytokines, IL10 and IFNg were not seen to be secreted following restimulation with *Na*-GST-1, but TNF and IL-2 were.

11- As total PBMC have been stimulated, are T cells the only or main TNF/IL-2 producing cells? Is there other population responding to the restimulation.

**Response**: As previously mentioned, CyTOF data analysis reveal that B cells are also responding to *Na*-GST-1 restimulation at day 194 by producing TNF.

12- The authors speculate in the discussion that the cytokine production in response to APR-1 stimulation could be due to a too low level of production, because of insufficient priming with NA-APR-1. Is CTLA-4 blockade enhancing cytokine production in response to APR-1 stimulation as well ? If yes, which cytokines are increased ?

**Response:** As the flow cytometry didn’t show an increase in cytokine response over time compared to baseline after APR-1 in vitro stimulation, it has not been checked whether blocking CTLA-4 would increase the antigen specific response. This would have been interesting and will be considered for future studies.

Conclusion: The conclusion is well written and support the data presented. Just a comment for the authors:

1- The authors state “No significant changes over time were detected for IFN-γ, TH2cytokines (IL-4/-5/-13) or IL-10 in response to Na-GST-1”  As type 2 responses are usually associated with protection against helminths, these results are quite surprising, could the authors expand further their comments on this in the discussion.

**Response**: These vaccine candidates were chosen to avoid any Th2 response induction. Previous vaccination with a hookworm vaccine candidate, Na-ASP-2, resulted in a generalized urticaria, leading to the suspension of the trial. This was the reason why new vaccine candidates (*Na*-APR-1 and *Na*-GST-1) were designed hoping to show minimal Th2 activity.

2- In particular on what could be expected in terms of efficacy of the treatment. To my knowledge it is unknown even in animal models how the immunisation with APR-1 is working. Are only the antibodies required or do the cellular immune response need to be polarised as well. The subclass of IgG in animal model for APR-1 have been proposed to be IgG1 and IgG4, which depends on IL-4 and IL-10 respectively for class switching.  A poor modified type 2 response could explain the low antibody response to APR-1. As immunization with GST-1 has been shown to give rise to IgG3 and IgG1, the type 2 cytokines might not be as required. Overall, it might be that it is not that much the dose of APR-1 that needs to be increased but maybe that another adjuvant is required.

**Response**: The reviewer raises an important question. However, as we do not detect a cellular response, it is difficult to assess if it is the correctly skewed response. The suggestion to use a different adjuvant might be needed to improve APR-1 responses and only then we can characterize that in more depth. We have added, a sentence to the Discussion (page 16, line 365) to consider this point as outlined below.

Abstract: (No comments)
